# Comparative analysis of the relationship of sarcopenia or sarcopenic obesity with functional impairment: A cross‐sectional study

**DOI:** 10.1002/ncp.70046

**Published:** 2025-10-05

**Authors:** Flavia Alves Gomes, Stephany Beatriz do Nascimento, Letícia Sabino Santos, Taynara de Sousa Rego Mendes, Roana Carolina Bezerra dos Santos, Maria Conceição Chaves de Lemos, Cláudia Porto Sabino Pinho

**Affiliations:** ^1^ Hospital of Clinics Federal University of Pernambuco Recife Brazil; ^2^ Brazilian Company of Hospital Services (EBSERH) Recife Brazil; ^3^ Department of Nutrition Federal University of Pernambuco Recife Brazil

**Keywords:** aging, body composition, muscle strength, obesity, sarcopenia

## Abstract

Growing evidence suggests that sarcopenic obesity (SO) may have a more pronounced effect on functionality compared with isolated sarcopenia, but research in this crucial area remains scarce. Therefore, this study aimed to evaluate whether the combination of sarcopenia and obesity is associated with increased functional impairment in hospitalized older adults. This is a cross‐sectional study involving hospitalized older patients. SO was defined as the simultaneous presence of obesity and sarcopenia. Obesity was determined based on a high body fat percentage obtained through bioelectrical impedance analysis, whereas both reduced muscle strength and mass identified sarcopenia. Functionality was evaluated using the Barthel Index and the gait speed test. Additional sociodemographic, clinical, nutrition, and behavioral data were assessed. A total of 176 patients were included in our study. The mean age was 69.8 ± 7.8 years. The frequency of sarcopenia was 37.5%, whereas SO was found in 17.6%. Barthel Index indicated that 64.2% of patients exhibited functional dependency, whereas 87.5% had a slow gait speed. Logistic regression analysis revealed that SO was independently associated with poor functionality by the Barthel Index (odds ratio [OR], 2.9; 95% confidence interval [CI], 1.1–8.5) and slow gait speed (OR, 3.3; 95% CI, 1.1–9.8). Patients with SO showed poorer functional capacity compared with those with obesity alone (*P* < 0.05), but not compared with those with sarcopenia alone (*P* > 0.05). In conclusion, we observe that SO was associated with diminished functionality but did not elevate the risk compared with sarcopenia alone.

## INTRODUCTION

Aging is a natural dynamic process associated with physiological modifications that occur in later years of life, promoting changes and/or redistribution of body composition (ie, decrease in muscle mass and increase in adipose tissues).^1^ Losing lean soft tissues is often linked to a decline in biological capacity. This process, characterized by muscle atrophy and fiber loss, begins in adulthood and continues throughout life, influenced by lifestyle and genetic factors. As a consequence, this muscle wasting may lead to abnormal conditions such as sarcopenia.^2^

Sarcopenia is now recognized as a disease characterized by a progressive skeletal muscle mass disorder, which is associated with poor clinical outcomes, including impaired quality of life.^2^, ^3^ Sarcopenia can be diagnosed by the reduction of muscle function and mass, also recognized as a multifactorial geriatric syndrome, linked to an increased risk of adverse outcomes.^3^

Sarcopenia may be accompanied by a relative or absolute body fat gain, potentially developing a condition known as sarcopenic obesity (SO). SO is a clinical condition in which there is a coexistence of excess fat mass with sarcopenia.^4^ According to the European Society for Clinical Nutrition and Metabolism (ESPEN) and the European Association for the Study of Obesity (EASO), the operational diagnosis of SO involves a two‐step process: (1) assessment of skeletal muscle function, including measurements of muscle mass and muscle strength; and (2) evaluation of body composition, typically performed using dual‐energy x‐ray absorptiometry, bioelectrical impedance analysis (BIA), or computed tomography, when clinically indicated for additional diagnostic purposes.^4^

Although it is well‐established that sarcopenia significantly impacts functionality, SO may have additional prognostic value in functional capacity, and its impact remains elusive. Growing evidence suggests that SO may have a more pronounced effect on functionality than isolated sarcopenia, but research in this crucial area remains scarce.^3^, ^4^

Understanding the negative clinical consequences of SO and its impact on functionality will provide valuable insights, aiding healthcare professionals working with the geriatric population and contributing to the scientific community's understanding of this relationship. Therefore, our study aimed to evaluate whether the combination of sarcopenia and obesity is associated with increased functional impairment in hospitalized older adults.

## METHODS

This is a cross‐sectional study involving hospitalized older individuals. Data collection occurred from March to September 2021 in a university hospital in Recife, Brazil. We included male and female patients (aged ≥60 years) admitted for hospitalization across various medical specialties, including cardiology, nephrology, gastroenterology, pulmonology, gynecology, urology, internal medicine, oncology, rheumatology, vascular medicine, and dermatology in Clinics Hospital of Federal University of Pernambuco, Brazil. Exclusion criteria were patients with physical and cognitive limitations, those who were completely bedridden, those in the immediate postoperative period of medium and large surgeries, individuals with mechanical protheses, those with chronic kidney disease undergoing dialysis, edema, or ascite, and those with joint abnormalities in the upper and lower limbs that prevented them from performing the gait speed (GS) and handgrip strength (HGS) tests.

Sample size was calculated using the STATCALC module of Epi Info software version 6.04. An estimated quantity of 400 hospitalizations of older individuals at the institution during the same period as the study conducted in the preceding year, a prevalence of SO of 23.4%,^5^ a standard error of 5%, and a 95% confidence interval (CI) were considered. This calculation determined a minimum sample of 163 patients to be evaluated. To account for potential losses, this number was increased by 10%, resulting in a total of 180 patients being recruited. The study was approved by the Ethics Committee on Research Involving Human Subjects, following Resolution 466/12 of the National Health Council (CAAE 63983322.7.0000.5208) and the Declaration of Helsinki and its subsequent amendments. All participants provided informed consent.

SO was diagnosed with the coexistence of obesity and sarcopenia. Obesity was characterized by a high body fat percentage (%BF), following diagnostic criteria established by the ESPEN and the EASO.^4^ %BF was assessed using BIA with a portable device, the Biodynamics model 310, which applies a current of 800 µA at a single frequency of 50 Hz. %BF values >40% for women and >30% for men were considered elevated.^4^

For BIA assessment, participants were instructed to fast for at least 4 h, abstain from caffeine for 24 h, wear light clothing, empty their bladders, and remove all metal objects before the examination. During the procedure, participants were positioned supine on a stretcher with their limbs slightly apart from their bodies. Four electrodes were placed in standard positions on the dorsum of the right hand and right foot. Demographic and anthropometric data, including age, height, weight, sex, and ethnicity, were entered into the BIA system.^6^

Muscle strength was assessed using HGS, measured with the JAMAR digital dynamometer. Low HGS was established based on the revised Sarcopenia consensus (European Working Group on Sarcopenia in Older People): HGS < 27 kg for men and HGS < 16 kg for women.^3^ Appendicular skeletal muscle mass (ASMM) was calculated using the equation by Sergi et al,^7^ which is based on weight and BIA‐derived resistance and reactance. Subsequently, the ASMM index (ASMI) was calculated using the formula^3^ ASMM/height^2^. Low ASMI was classified based on the Brazilian population cutoffs: ≤7.7 kg/m^2^ for men and ≤5.62 kg/m^2^ for women.^8^

All evaluations were conducted up to 48 h after hospital admission. Using the parameters aforementioned, participants were categorized into four subgroups based on their nutrition phenotype: isolated sarcopenia, isolated obesity, SO, and nonobese and/or nonsarcopenic.

Functionality was assessed using the Barthel Index (BI) and the GS test. The BI is an assessment tool for activities of daily living (ADLs), covering self‐care, mobility, locomotion, and elimination activities. The maximum score is 100, and the lowest is 0, with questions scored based on the degree of independence, depending on the time and assistance required. BI assesses aspects such as feeding, personal hygiene, independence in dressing, bowel and bladder continence, independence in using the bathroom, locomotion, and mobility.^9^ Patients with a maximum score (100 points) were considered independent; a score of 76–99 was classified as mild dependence; 51–74 points indicated moderate dependence; 26–50 points signified severe dependence; and <25 was classified as total or severe dependence.^9^ For analysis, the classification was dichotomized into functional independence (independent and mildly dependent) and functional dependence (moderate to severe dependence).^10^

The GS test was conducted following the protocol outlined by the International Academy on Nutrition and Aging and involved having the individuals walk at their usual pace over a 4‐m distance on a flat surface. The assessments were performed twice, and the time taken for the shorter of the two trials was used as the reference. GS was considered slow when the speed was <0.8 m/s.^11^

Sociodemographic and clinical data were collected, including age, sex, education level (in years of schooling), and clinical diagnosis of hospitalization. Clinical diagnoses were categorized into two groups: malignancies (all types of cancer) and nonmalignant organic disorders (digestive and endocrine disorders, diseases of the nervous system, infectious diseases, respiratory diseases, autoimmune disorders, and renal and ureteral diseases).^10^

Nutrition status was assessed using the body mass index (BMI), classified according to Lipschitz's criterion.^12^ Physical activity level was evaluated using the International Physical Activity Questionnaire 2001 in its short version, and physical activity level was dichotomized into sufficiently active (>150 min of activity per week) and insufficiently active or sedentary (<150 min of activity per week).^13^

Data were analyzed using the statistical software SPSS version 13.0 (SPSS Inc, Chicago, IL, USA). Bivariate analysis was conducted, employing either Yates' correction test for 2 × 2 tables or Fisher's exact test, as appropriate. To explore factors associated with low functionality, a binary logistic regression model was constructed. Multicollinearity among independent variables was assessed using the Variance Inflation Factor statistics (with values > 0.10 and <3 considered acceptable) and tolerance. Two separate regression models were developed, one for sarcopenia and another for SO, because of collinearity concerns. Variables that exhibited a *P* value <0.20 in the univariate analysis were included in the multivariate analysis. Adjusted odds ratios (ORs) with their corresponding 95% CIs were calculated using the Wald method. Model goodness of fit was evaluated using the Hosmer and Lemeshow test. Post hoc 2 × 2 comparisons using the chi‐square test were conducted to identify differences between the studied phenotypes of obesity and sarcopenia. Analyses with a significance level of <0.05 were considered statistically significant for the final model.

## RESULTS

A total of 180 eligible patients were initially recruited, but 4 were removed because of inconsistent data (*n* = 2) or refusal to participate (*n* = 2). The final sample included 176 patients (mean age, 69.8 ± 7.8 years; 54.5% men). Most patients had a low educational level (≤9 years of schooling) (71.6%), had nonmalignant diseases (63.6%), and were sedentary (80.1%). A total of 52.3% of the sample had excess weight by BMI, whereas 46.6% had excess body fat estimated by BIA. Sarcopenia alone was found in 37.5%, whereas SO was found in 17.6% of the sample. BI demonstrated that 64.2% of the patients had functional dependence, and 87.5% had slow GS (Table 1).

**Table 1:** Demographic, clinical, behavioral, nutrition, and functional characteristics of hospitalized geriatric patients (*N* = 176).

Factors	*n*	%
Male sex	96	54.5
Age, y		
60–69	102	58.0
70–79	54	30.7
≥80	20	11.3
Schooling (≤9 years of study)	126	71.6
Nonmalignant diagnosis	112	63.6
Sedentarism	141	80.1
BMI, kg/m^2^		
Underweight	15	8.5
Eutrophy	69	39.2
Excessive weight	92	52.3
High body fat, %	82	46.6
Functional dependence^a^	113	64.2
Slow gait speed	154	87.5
Phenotypes		
Sarcopenia	35	19.9
Obesity	51	29.0
Sarcopenic obesity	31	17.6
Without obesity and without sarcopenia	59	33.5

Abbreviation: BMI, body mass index.

^a^ Barthel Index.

Bivariate analysis demonstrated that low functionality (BI) increased with age progression and was more frequent among women, sedentary individuals, those with sarcopenia alone, and individuals with SO (*P* < 0.05). Low functionality by GS was more frequent among women, individuals aged 80 years or older, sedentary individuals, and those with sarcopenia alone (*P* < 0.05) (Table 2).

**Table 2:** Analysis of demographic, clinical, behavioral, and nutrition factors associated with reduced functionality according to the Barthel Index and gait speed in hospitalized geriatric individuals (*N* = 176).

Factors	Barthel Index, functional dependence, *n* (%)	*P* value	Slow gait speed, *n* (%)	*P* value
Male sex		0.036^a^		0.022^b^
Men	55 (57.3)		79 (82.3)	
Women	58 (72.5)		75 (93.8)	
Age, y		0.029^a^		0.047^a^
60–69	58 (56.9)		89 (87.3)	
70–79	38 (70.4)		45 (83.3)	
≥80	17 (85.0)		20 (100.0)	
Years of study		0.075^a^		0.129^a^
≤9	86 (68.3)		113 (89.7)	
>9	27 (54.0)		41 (82.0)	
Diagnosis		0.494^a^		0.636^a^
Nonmalignant	74 (66.1)		99 (88.4)	
Malignancies	39 (60.9)		55 (85.9)	
Physical activity		0.011^a^		<0.001^a^
Sedentarism	97 (68.8)		131 (92.9)	
Active	16 (45.7)		23 (65.7)	
BMI, kg/m²		0.739^a^		0.121^a^
Underweight	44 (63.8)		64 (92.8)	
Eutrophy	11 (73.3)		14 (93.3)	
Excessive wieght	58 (63.0)		76 (82.6)	
Body fat, %		0.458^a^		0.732^a^
Normal	58 (61.7)		83 (88.3)	
High	55 (67.1)		71 (86.6)	
Sarcopenia		0.004^a^		0.002^b^
No	62 (56.4)		90 (81.8)	
Yes	51 (77.3)		64 (97.0)	
Sarcopenic obesity		0.012^b^		0.067^b^
No	87 (60.0)		124 (85.5)	
Yes	26 (83.9)		30 (96.8)	

*Note*: Two‐tailed *P* value.

Abbreviation: BMI, body mass index.

^a^ Pearson chi‐square.

^b^ Fisher's exact test.

Binary logistic regression model demonstrated that being women (OR, 2.0; 95% CI, 1.1–4.1), age (OR, 1.1; 95% CI, 1.0–1.2), and having sarcopenia (OR, 2.5; 95% CI, 1.2–5.2) were independently associated with low functionality by BI. Additional analysis demonstrated being women (OR, 3.0; 95% CI, 1.1–9.4), sedentary (OR, 4.8; 95% CI, 1.7–13.8), and having sarcopenia (OR, 74; 95% CI, 1.6–35.2) remained independently associated with low functionality by GS (Table 3).

**Table 3:** Binary logistic regression of sarcopenia and factors associated with reduced functionality in hospitalized geriatric individuals (*N* = 176).

Factors	Functional dependence (BI)		Slow gait speed	
	Adjusted OR (95% CI)	*P* value^b^	Adjusted OR (95% CI)	*P* value^b^
Sex				
Men^a^	1.0		1.0	
Women	2.0 (1.1–4.1)	0.049	3.0 (1.1–9.4)	0.047
Age, y	1.1 (1.0–1.2)	0.018	1.0 (0.9–1.1)	0.901
Years of study				
≤9	1.5 (0.8–3.2)	0.219	1.5 (0.5–4.3)	0.431
>9^a^	1.0		1.0	
Physical activity				
Sedentarism	1.6 (0.7–3.7)	0.237	4.8 (1.7–13.8)	0.003
Active^a^	1.0		1.0	
Sarcopenia				
No sarcopenia^a^	1.0		1.0	‐
Sarcopenia	2.5 (1.2–5.2)	0.018	7.4 (1.6–35.2)	0.012

Abbreviations: BI, Barthel Index; CI, confidence interval; OR, odds ratio.

^a^ Reference groups: men, years of study >9, active, no sarcopenia.

^b^ Wald test.

The adjusted models that included SO showed that age (OR, 1.1; 95% CI, 1.0–1.2) and SO (OR, 2.9; 95% CI, 1.1–8.5) were independently associated with low functionality by BI. Poor physical activity status (OR, 4.9; 95% CI, 1.7–14.0) and SO (OR, 3.3; 95% CI, 1.1–9.9) were independently associated with poor performance based on the GS test (Table 4).

**Table 4:** Binary logistic regression of sarcopenic obesity and factors associated with reduced functionality in hospitalized geriatric individuals (*N* = 176).

Factors	Functional dependence (BI)		Slow gait speed	
	Adjusted OR (95% CI)	*P* value^b^	Adjusted OR (95% CI)	*P* value^b^
Sex				
Men^a^	1.0		1.0	
Women	1.9 (0.9–3.7)	0.084	2.8 (0.9–8.8)	0.073
Age, y	1.1 (1.0–1.2)	0.019	1.0 (0.9–1.1)	0.908
Years of study				
≤9	1.8 (0.9–3.6)	0.126	1.0	
>9^a^	1.0		1.7 (0.6–4.9)	0.290
BMI, kg/m^2^	1.0 (0.9–1.1)	0.866	0.9 (0.8–1.0)	0.104
Physical activity				
Sedentarism	1.6 (0.7–3.6)	0.277	4.9 (1.7–14.0)	0.003
Active^a^	1.0		1.0	
Sarcopenic obesity				
No SO^a^	1.0		1.0	
SO	2.9 (1.1–8.5)	0.048	3.3 (1.1–9.8)	0.059

Abbreviations: BI, Barthel Index; BMI, body mass index; CI, confidence interval; OR, odds ratio; SO, sarcopenic obesity.

^a^ Reference groups: men, years of study >9, active, no SO.

^b^ Wald test.

The comparative analysis of functionality according to obesity and sarcopenia phenotypes indicated a significant difference in low functionality rates among the four evaluated phenotypes (*P* < 0.05). When the analyses were stratified by group, individuals with SO showed significantly lower functionality, as measured by both the BI and GS, compared with those with obesity alone (*P* < 0.05) and those without either sarcopenia or obesity (*P* < 0.05). However, their functionality was similar to that of individuals with sarcopenia alone (*P* < 0.05). In other words, the presence of SO increased the likelihood of low functionality compared with those with obesity alone but did not further increase the risk of functional dependence compared with individuals who were already sarcopenic (*P* > 0.05) (Table 5).

**Table 5:** Comparative analysis of functionality according to obesity and sarcopenia phenotypes in hospitalized geriatric patients (*N* = 176).

Functionality	Sarcopenia	Obesity	Sarcopenic obesity	Without sarcopenia and obesity	*P* value
Barthel Index, *n* (%)					0.030^a^
Functional independence	10 (28.5)	22 (43.1)	5 (16.1)	26 (44.1)	
Functional dependence	25 (71.5)	29 (56.9)	26 (83.9)	33 (55.4)	
Gait speed, *n* (%)					0.032^b^
Normal	1 (2.9)	10 (19.6)	1 (3.2)	10 (16.9)	
Slow	34 (97.1)	41 (80.4)	30 (96.8)	49 (83.1)	

*Note*: *P* value for 2 × 2 comparison (Pearson chi‐square) using the Barthel Index:
Isolated sarcopenia group vs group without sarcopenia and obesity: *P* = 0.135

Isolated sarcopenia group vs isolated obesity group: *P* = 0.170
Isolated sarcopenia group vs SO group: *P* = 0.229

SO group vs group without sarcopenia and obesity: *P* = 0.008*
SO group vs isolated obesity group: *P* = 0.012*

Isolated obesity group vs group without sarcopenia and obesity: *P* = 0.458

*P* value for 2 × 2 comparison (Pearson chi‐square) using gait speed test:
Isolated sarcopenia group vs group without sarcopenia and obesity: *P* = 0.036*

Isolated sarcopenia group vs isolated obesity group: *P* = 0.020*
Isolated sarcopenia group vs SO group: *P* = 0.723

SO group vs group without sarcopenia and obesity: *P* = 0.050*
SO group vs isolated obesity group: *P* = 0.032*

Isolated obesity group vs group without sarcopenia and obesity: *P* = 0.732

Abbreviation: SO, sarcopenic obesity.

^a^ Pearson chi‐square test.

^b^ Fisher's exact test.

* indicates statistically significant differences.

## DISCUSSION

In this study, we investigated the relationship between sarcopenia, SO, and functionality in a group of older adults admitted for hospitalization. Functionality was assessed using the BI and GS test, revealing that sarcopenia and SO were independently associated with low functionality, significantly increasing the likelihood of poorer performance. The synergy between obesity and sarcopenia, characteristic of SO, heightened the risk of functional impairment compared with obesity alone but did not further decrease functionality compared with individuals with sarcopenia alone.

High rates of sarcopenia and SO were observed in the study. Sarcopenia affected more than one‐third of the population (37.5%), highlighting the importance of systematically assessing these conditions in hospitalized older individuals. Our result aligns with findings from a meta‐analysis conducted by Ligthart‐Melis et al, which described a prevalence of 37% in hospitalized patients aged ≥65 years.^14^ Another study that assessed 639 individuals with a mean age of 80.9 years reported sarcopenia rates of 12.4% in women and 23.5% in men.^15^ Globally, the prevalence varies from 10% to 27% among individuals aged ≥60 years.^14^

Although identified in a smaller proportion, the prevalence of SO (17.6%) was higher than previously reported.^16^ Santos et al identified a SO prevalence of 4.4% in Brazilian older individuals aged ≥65 years living in the community, using BMI as the parameter for assessing obesity.^17^ Pillat et al likewise reported a prevalence of SO in 4.4% of their population (206 older individuals using primary care services in southern Brazil, considering %BF as the method for assessing obesity).^18^

It is possible that the higher prevalence in our sample can be associated with the clinical characteristics of our population (ie, older patients from a hospital setting). Hospitalization in older individuals is a risk factor for muscle wasting and functional decline. These consequences arise from physical stress resistance, such as trauma or illness, as well as factors like malnutrition, sleep deprivation, polypharmacy, and excessive rest.^10^ Additionally, we used %BF as a parameter for assessing obesity, which is one of the most accurate measures for evaluating adiposity.^19^, ^20^

Low functionality was also notably high, as indicated by both methods (64.2% based on the BI and 87.5% based on the GS). The study by Paixão et al reported a 34% prevalence of reduced functionality (using the Katz index) among 241 older individuals aged ≥80 years receiving primary care in southeastern Brazil.^21^ These results are consistent with the findings of Mancini et al, who conducted a cross‐sectional study involving institutionalized geriatric individuals aged ≥60 years in long‐term care facilities and reported a 57% prevalence of reduced functionality based on physical assessment protocols.^22^ The notable differences in our findings can be attributed to the characteristics of our studied population (older individuals recruited in a hospital setting), the specific functionality assessment tools, and the subjective nature of the physical test protocols.

In our study, functionality was qualitatively assessed using the GS test, an accessible and simple method for application in older individuals. This method is recommended by the European Consensus on Sarcopenia^3^ because of its practicality and ability to predict negative outcomes associated with sarcopenia. Although there are few studies on its applicability in hospitalized older individuals, Martinez et al reinforced its feasibility by applying it to hospitalized individuals, confirming the method's reproducibility and safety.^16^ Additionally, our study adopted self‐reporting of ADLs through the BI. BI is frequently used as an assessment tool because it covers various levels of disabilities and different profiles of geriatric individuals, providing robust results by estimating functional capacity.^9^

Associations between sarcopenia and poorer functional outcomes have been well documented in the scientific literature, but few studies have explored the synergistic effects of obesity and sarcopenia on functional aspects.^3^, ^23^ Our findings demonstrated that the SO phenotype was similarly a powerful independent factor related to reduced functionality, tripling the odds of poor functionality. An exploratory study with 234 Japanese older individuals showed that individuals with SO had worse results in physical fitness according to the GS test.^24^

Our additional results showed that SO did not increase the risk of functional dependency when compared with sarcopenia alone. This finding is consistent with the study by Santos et al, which assessed women aged 80 and older and did not find a lower likelihood of mobility reduction in those with SO compared with isolated obese or sarcopenic individuals.^25^ These findings are also supported by the results of Bahat et al.^26^ The authors analyzed 1468 Turks aged 60 and older and found that individuals with SO had impaired functional measures but did not experience a greater deterioration in ADLs compared with those with isolated sarcopenia.^26^

However, Batsis et al conducted a study with 4984 older individuals in the United States and identified a strong association between SO and functional impairment, with a greater impact on physical capacity compared with isolated sarcopenia.^27^ Another study involving 1373 Brazilian geriatric individuals showed that patients with SO had worse functional performance in terms of ADL when compared with isolated obesity.^17^ These findings are consistent with our results, which showed that SO increased the likelihood of impaired functional measures compared with obesity alone, but not compared with sarcopenia alone. This suggests that sarcopenia may have a greater impact on functional impairment than obesity.

It is still debated whether the outcomes of SO are multiplicative or if obesity in this population may have protective effects.^28^ In older adults, increased body adiposity may provide protection against certain adverse outcomes, a phenomenon known as the obesity paradox.^29^ Our data did not show differences between those with obesity alone and individuals without sarcopenia or obesity, indicating that isolated obesity was not particularly detrimental to functional impairment.

Previous studies have suggested a protective role of obesity in functional measures. In a study involving 4944 Polish adults aged 65 and older, overweight and obesity were not associated with deterioration in ADLs, and a higher BMI was associated with better ADL scores in adjusted analyses.^30^ Several explanations have been proposed to indicate why obesity may not be detrimental to the functionality of older adults: (1) overweight or obesity may be associated with a better supply of vitamins and other nutrients, contributing to improved overall physiological function; (2) obese individuals tend to have higher levels of estrogen, which has positive effects on muscle mass; and (3) there may be protection against osteoporotic fractures because higher BMI is associated with greater bone mineral density, and the cushioning effect of fat provides protection against fractures and falls.^26^, ^30^ It is relevant to note that the evidence supporting the “obesity paradox” in aging remains controversial, with this supposed protective effect disappearing in the context of SO. Thus, whether the coexistence of sarcopenia and obesity increases the risk of adverse events requires substantial exploration.^31^

Our study is not without limitations. Its cross‐sectional design does not allow for establishing a cause‐and‐effect relationship between any of the body phenotypes and functional measures. It is not possible to assert whether sarcopenia and SO led to low functionality or vice versa. Additionally, single‐center recruitment restricts the sample's representativity and the generalizability of the results to other populations of older individuals, limiting its external validity. Additionally, this study did not examine the history of falls, previous hospitalizations, or clinical follow‐up of the older adults, factors that could influence sarcopenia estimates. Outcome data, such as length of hospital stay and mortality rates, could also contribute to the discussion on the ability of functionality and SO to predict adverse outcomes. Future studies could explore these phenotypes, also considering emotional and cognitive aspects because they may affect both nutrition status and functionality.

Another relevant aspect concerns BIA‐related limitations. Hydration status and disease‐related alterations may compromise the reliability of this method, particularly in hospitalized patients, requiring caution to interpret our results. It is also important to acknowledge that we used only BMI to “assess” nutrition status and did not apply more detailed tools such as the Mini Nutritional Assessment (MNA). Although BMI has limitations, we used age‐specific cutoff points for older adults, which better reflect changes in body composition occurring with aging. These cutoffs have been shown to correlate more closely with MNA results than the standard BMI values recommended by the World Health Organization (WHO).^12^, ^32^

Because of the relatively small sample size, this study was also limited to performing multinomial modeling for direct comparison of body phenotypes. Future studies with larger samples should consider this approach to better explore these differences.

## CONCLUSION

Sarcopenia and SO were common among hospitalized geriatric individuals, and both conditions were independently associated with poor functionality. However, the coexistence of both obesity and sarcopenia (SO) did not increase the likelihood of reduced functionality when compared with individuals with isolated sarcopenia but did worsen functionality results compared with isolated obesity. Further studies are needed to investigate the synergistic effect of these risk conditions on reduced functionality to reach more definitive conclusions.

## AUTHOR CONTRIBUTIONS

Flavia Gomes and Cláudia Pinho equally contributed to the conception and design of the research. Stephany Nascimento, Letícia Santos, Tayanara Mendes, and Roana Santos contributed to the acquisition of the data. Cláudia Pinho and Flavia Gomes contributed to the analysis and interpretation of the data. Flavia Gomes drafted the manuscript. Cláudia Pinho and Maria Conceição Chaves contributed to the critical review of the manuscript. All authors revised the manuscript, agree to be fully accountable for ensuring the integrity and accuracy of the work, and read and approved the final manuscript.

## CONFLICT OF INTEREST STATEMENT

None declared.
